# Implementation of comprehensive services as a mediator and race and ethnicity as a moderator of access and retention in addiction health services

**DOI:** 10.1186/1940-0640-10-S1-A18

**Published:** 2015-02-20

**Authors:** Erick G Guerrero, Karissa Fenwick, Christine Grella, Thomas D'Aunno

**Affiliations:** 1 School of Social Work, University of Southern California, Los Angeles, CA, 90089, USA; 2 Integrated Substance Abuse Programs, Department of Psychiatry & Biobehavioral Sciences, University of California, Los Angeles, Los Angeles, CA, 90025, USA; 3 Mailman School of Public Health, Columbia University, New York, NY, 10032, USA

## Background

Increasing evidence has suggested that well-resourced programs are more likely than low-resourced programs to deliver comprehensive care to individuals with co-occurring conditions and achieve greater client access and retention. But there is limited understanding of the service delivery process that allows high-capacity programs to reduce their wait times for treatment entry and their rates of dropout.

## Purpose

To examine the indirect association of program capacity factors with wait time and retention via mental health and HIV prevention services in publicly funded addiction health services (AHS).

## Methods

We conducted multilevel cross-sectional analysis of 108 publicly funded program data merged with Los Angeles County Participant Reporting System data from 2010 to 2011 for 13,478 adults entering AHS. Multilevel negative binomial regression models were used to test direct and indirect relationships between program capacity and days to enter treatment (wait time) and days in treatment (retention).

## Findings

Findings show that compared to Whites, Latinos and African Americans served in high-capacity programs reported shorter wait times and higher retention rates. As hypothesized, the role of HIV testing and mental health service coordination played an indirect role in the relationship between program capacity and shorter wait times.

## Conclusions

Findings suggest that coordinated comprehensive services in AHS may contribute to reduction of outcome disparities in access to care. Delivery of comprehensive care may allow programs to develop effective networks to reduce wait time. Implications for health-care reform implementation and program planning are discussed.

